# The Prevalence and Molecular Biology of *Staphylococcus aureus* Isolated from Healthy and Diseased Equine Eyes in Egypt

**DOI:** 10.3390/antibiotics11020221

**Published:** 2022-02-10

**Authors:** Amin Tahoun, Helmy K. Elnafarawy, Hanem El-Sharkawy, Amira M. Rizk, Mohammed Alorabi, Ahmed M. El-Shehawi, Mohamed A. Youssef, Hussam M. M. Ibrahim, Sabry El-Khodery

**Affiliations:** 1 Department of Animal Medicine, Faculty of Veterinary Medicine, Kafrelshkh University, Kafrelsheikh 33511, Egypt; 2 Department of Internal Medicine and Infectious Diseases, Faculty of Veterinary Medicine, Mansoura University, Mansoura 35516, Egypt; helmykamal@mans.edu.eg (H.K.E.); mohamed.youssef@mans.edu.eg (M.A.Y.); dr_hussamhabosha@yahoo.com (H.M.M.I.); khodery@mans.edu.eg (S.E.-K.); 3 Department of Poultry and Rabbit Diseases, Faculty of Veterinary Medicine, Kafrelsheikh University, Kafrelsheikh 33511, Egypt; hanem_amin@yahoo.com; 4 Department of Bacteriology, Mycology and Immunology, Faculty of Veterinary Medicine, Benha University, Benha 13518, Egypt; dr_az80@yahoo.com; 5 Department of Biotechnology, College of Science, Taif University, P.O. Box 11099, Taif 21944, Saudi Arabia; maorabi@tu.edu.sa (M.A.); a.elshehawi@tu.edu.sa (A.M.E.-S.)

**Keywords:** antibiotics, antimicrobial resistance, risk factors, equines, virulence, toxins, oxacillin, MRSA

## Abstract

This work aimed to characterize *S. aureus* isolates from the eyes of healthy and clinically affected equines in the Kafrelsheikh Governorate, Egypt. A total of 110 animals were examined for the presence of *S. aureus*, which was isolated from 33 animals with ophthalmic lesions and 77 healthy animals. We also investigated the antimicrobial resistance profile, oxacillin resistance mechanism, and the major virulence factors implicated in many studies of the ocular pathology of pathogenic *S. aureus*. The association between *S. aureus* eye infections and potential risk factors was also investigated. The frequency of *S. aureus* isolates from clinically affected equine eyes was significantly higher than in clinically healthy equids. A significant association was found between the frequency of *S. aureus* isolation from clinically affected equine eyes and risk factors including age and season but not with sex or breed factors. Antimicrobial resistance to common antibiotics used to treat equine eyes was also tested. Overall, the isolates showed the highest sensitivity to sulfamethoxazole (100%) and the highest resistance to cephalosporin (90.67%) and oxacillin (90.48%). PCR was used to demonstrate that *mecA* was present in 100% of oxacillin- and β-lactam-resistant *S. aureus* strains. The virulence factor genes *Spa* (x region), *nuc*, and *hlg* were identified in 62.5%, 100%, and 56%, of isolates, respectively, from clinically affected equines eyes. The severity of the eye lesions increased in the presence of γ-toxin-positive *S. aureus*. The phylogenetic tree of the *Spa* (x region) gene indicated a relationship with human reference strains isolated from Egypt as well as isolates from equines in Iran and Japan. This study provides insight into the prevalence, potential risk factors, clinical pictures, zoonotic potential, antimicrobial resistance, and β-lactam resistance mechanism of *S. aureus* strains that cause eye infection in equines from Egypt.

## 1. Introduction

Bacterial infection of the eye causes diseases, including conjunctivitis, keratitis, endophthalmitis, and corneal ulcers, of which the bacteria *Staphylococcus aureus* is considered a major causative agent [1]. The bacterial community that inhabits the corneal and conjunctival spaces of healthy horses includes non-pathogenic and opportunistic Gram-positive (G +ve) bacteria [2]. The robust immune system of the horse cornea reduces the possibility of infection; however, opportunistic bacteria such as *S. aureus* exploit antecedent trauma or pre-existing disease to overcome the host immune defense [3]. Whenever the natural barriers of the host are lowered, *S. aureus* produces a wide range of purulent and/or toxin-mediated diseases [4]. The development of methicillin resistance in some *S. aureus* lineages and its fast spread in recent decades is a challenge to human and animal healthcare practitioners [5].

Horse eye diseases and their associated complications are serious health threats to the animal, as they affect its quality of life, its value, and reduce its use for athletic and show purposes. Threatening eye infections that may disrupt horse training and potentially disqualify the animal from competition may necessitate long and expensive treatment courses [6]. Such infections result in a huge economic loss to the horse sports and trading industry, which is estimated at a million dollars a year in the USA [7]. Since *S. aureus* is able to accommodate various environments and develop diverse new antibiotic resistance mechanisms, it is a predominant bacterial cause of endemic human and animal diseases worldwide [8]. Additionally, due to the close relationship between horse and human, especially in sports, there is a high risk for zoonotic transmission to human caregivers. The bacterial community that inhabits the cornea and conjunctiva of healthy horses includes nonpathogenic and opportunistic Gram-positive (G +ve) bacteria [2]. Furthermore, another study identified methicillin-resistant *S. aureus* (MRSA) from a donkey conjunctival swab [9].

The probable presence of MRSA in the normal ocular microbiota of healthy horses increases the chance of opportunistic MRSA infections and increases the likelihood of horse eye destruction if it is not diagnosed and treated quickly, which is complicated by the lack of treatment options for MRSA infections. MRSA has been repeatedly isolated from individuals in close contact with infected animals [10]. Therefore, horses, in addition to other pets, including dogs and cats, are often regarded as possible MRSA reservoirs [11,12]. This assumption was strengthened with the detection of genetically related MRSA isolates from horses and their human companions [13]. A large new body of evidence indicates an increase in MRSA infections in animals, including horses, that have close contact with human companions [14,15,16]. Moreover, humans can also be a source of MRSA infection for horses, as MRSA strains can also be transferred from humans to animals [14].

The prevalence of MRSA infection is increasing worldwide with high morbidity and mortality, and since it has multidrug resistance, there are narrow therapeutic options, which makes it a significant health concern [1,17]. Geographical differences between MRSA lineages worldwide have been proposed due to its ability to colonize and infect a wide host range and then circulate within distinct ecological environments [18,19]. This may weaken the efficacy of a general protocol for management. A spatiotemporal relationship between the isolates was also assumed [18]. Therefore, it is highly probable that the similarity between MRSA strains infecting humans and animals is high within each locality. Recently, an *S. aureus* lineage belonging to sequence type (ST) 398 was indicted as the predominant *S. aureus* strain infecting animals in Europe; due to its continuous acquisition of new antibiotic-resistance and virulence determinants, it was described as a serious public health concern [20]. The study of *S. aureus* strains in our country to characterize the virulence factors, drug-resistance patterns, and prevalence of regional strains may be required to enable the construction of region-specific plans for the proper management, control, and treatment of *S. aureus*.

To the best of our knowledge, a major study has not been conducted to characterize the predominant MRSA strains across Egypt. This study was designed to investigate the epidemiological, microbial, and molecular patterns of *S. aureus* isolated from clinically healthy and clinically affected equine eyes in the Kafrelsheikh governorates of Egypt. We report the resistance profiles of pathogenic *S. aureus* isolates to antibiotics and their associated mechanism(s). We also conducted a phylogenetic analysis of the *Spa* x region of pathogenic *S. aureus* strains to describe the zoonotic potential of these isolates within the region based on their relationship to previous isolates.

## 2. Material and Methods

### 2.1. Study Design

This survey targeted equines from December 2019 to June 2020 in the Kafrelsheikh province of northern Egypt. The survey effort was applied proportionally across the province of Kafrelsheikh based on the number of horses and donkeys in each town or city. The total number of equines in the study was 13,000, with a sample size of 0.85% using survey design and analysis software (survey toolbox program), which was used for the random selection of 220 eye swap samples from 110 equines, and several animals were sampled depending on the size of the herd. A total of 110 animals, 33 with ophthalmic lesions and 77 healthy, were selected for the study. The animals were randomly selected from 14 village herds. The herd effect was considered in logistic regression analysis.

All horses were fed a concentrated diet with berseem in the winter and hay in the summer. *S. aureus* were isolated from clinically healthy and clinically affected equine eyes.

Ophthalmic examination procedures included a general inspection of the eyes as well as detailed ocular examination [21]. All experiments and animal procedures were conducted in accordance with local guidelines following the approval of all experimental procedures by the local ethical committee of infectious diseases at the Faculty of Veterinary Medicine, Kafrelsheikh University, Egypt (ethical approval number KFS-2019/1). The samples were collected after obtaining permission from the farm owners, farm managers, or herdsmen.

### 2.2. Sample Collection

A total of 220 swabs were collected from 110 equines from several locations in the Kafrelsheikh province. A swab from the ocular conjunctiva of each was collected from the inferior conjunctival fornix of both eyes, without touching the eyelashes or eyelids. All samples were collected before the animals received any antibiotics or anesthetics [21]. The collected swabs were placed directly into sterile test tubes containing tryptone soya broth (Oxoid, Altrincham, UK) as an enrichment medium. The samples were kept on ice in power-cooled boxes until they were transported to the laboratory, within 5 h of collection, for further bacteriological examination.

### 2.3. Isolation and Identification of Staphylococcus Species

The samples inoculated in enrichment broth were incubated overnight at 37 °C and then a loopful was streaked on mannitol salt agar medium (Lab. Lemco 400, Worthing, UK). A colony from each plate suspected of being *S. aureus*, based on agar appearance, was picked and streaked on Baird Parker agar plates then incubated for 16 to 18 h at 37 °C. All *S. aureus* isolates were identified based on culture characteristics on Baird Parker agar with mannitol salt. The coagulase test was also performed to confirm *S. aureus* isolation. All *S. aureus* isolates were biochemically confirmed using the API 20NE system (BioMerieux, Marcy-l’E’ toile, France) and molecularly confirmed by PCR with staphylococcal 16s RNA primers to confirm the Staphylococcal genus and with the *nuc* gene to confirm the species *S. aureus* (Table 1).

### 2.4. Antibiotic Susceptibility Testing

The antibiotic sensitivity test was performed for all pathogenic *S. aureus* isolates using the Kirby–Bauer disk diffusion method [21]. Each isolate was inoculated in Mueller–Hinton broth and incubated for 24 h. The bacterial suspension was adjusted to match the 0.5 McFarland standard (approximately 1–2 × 10^8^ CFU/mL for American Type Culture Collection 25922 *E. coli*) using sterile saline solution. Each saline suspension was spread onto the surface of Mueller–Hinton agar plates with a sterile swab, and paper disks impregnated with antibiotics were dispensed onto the surfaces of Mueller–Hinton agar plates that were at least 24 mm apart from the center of each other using a multi-disk dispenser. The used antibiotic disks were 30 μg tetracycline, 1 μg oxacillin, 5 μg rifampicin, 10 μg ampicillin, 25 μg sulfamethoxazole/trimethoprim, 10 μg gentamicin, 25 μg streptomycin, 30 μg chloramphenicol, and 10 μg cephalosporin (Oxoid, UK). The plates were then incubated at 37 °C for 24 h. The diameters of the inhibition zones were measured using a caliper and interpreted using standard break points according to the European Committee on Antimicrobial Susceptibility Testing [22] to classify antibiotics as susceptible, intermediate, and resistant. The antimicrobial disks were selected based on the active principles most frequently used in the ophthalmic treatment routine of horses. Topical gentamicin and chloramphenicol are antibiotics indicated for the preventive treatment of corneal ulcers in horses [23,24]; chloramphenicol is still effective against MRSA strains [25].

### 2.5. DNA Extraction and PCR

Colonies of *S. aureus* were grown in 5 mL of tryptone soya broth at 37 °C with shaking for 16 h. DNA was extracted from these samples using the QIAamp DNA Mini kit (Qiagen, Germantown, MD, USA) following the manufacturer’s protocol. The extracted DNA was used for PCR amplification of the Staphylococcal 16s RNA region for the genus as well as the *S. aureus spa* (x region), *nuc*, *mecA*, and *hlg* genes. Cycling conditions and the gene-related primers are described in Table 1.

### 2.6. Sequencing and Phylogenetic Analysis

Three PCR samples were selected from *S. aureus spa* (x region) gene-positive samples (one sample from each animal species: Arabian horse, draft horse, and donkey) and their nucleotide sequences were determined to confirm the accuracy of the amplified gene. In this regard, the PCR products were purified using the QIAquick PCR product extraction kit (Qiagen). The Bigdye Terminator V3.1 cycle sequencing kit (Perkin-Elmer, Applied Biosystems, Foster City, CA, USA) was used for the sequence reaction and purified using a Centri-Sep spin column. DNA sequences were obtained with an Applied Biosystems3130 genetic analyzer (HITACHI, Tokyo, Japan). A BLAST^®^ analysis (Basic Local Alignment Search Tool) [26] was initially performed to establish sequence identity with GenBank accessions. The phylogenetic tree was created with the MegAlign module of LasergeneDNAStar version 12.1 [27], and the phylogenetic analyses were performed using maximum likelihood, neighbor joining, and maximum parsimony in MEGA6 [28]. The nucleotide sequences of the *S. aureus Spa* (x region) gene fragment from the three selected samples were then deposited in GenBank (NCBI) with accession numbers MZ005310/Arab horse/Egypt/SA-AH/2019, MZ005311/Draft horse/Egypt/SA-DH/2019, and MZ005312/Donkey/Egypt/SA-D/2019.

**Table 1:** Primer sequences, target genes, amplicon sizes, and cycling conditions.

Target Gene	Primer Sequences	Amplified Segment (bp)	Primary Denaturation	Amplification (35 Cycles)			Final Extension	Reference
				Secondary Denaturation	Annealing	Extension		
*spa* (x region)	For CAA GCA CCA AAA GAG GAA	Variable	94 °C 5 min.	94 °C 30 s.	60 °C 40 s.	72 °C 40 s.	72 °C 7 min.	[29]
	Rev CAC CAG GTT TAA CGA CAT							
*nuc*	For ATATGTATGGCAATCGTTTCAAT	395	94 °C 5 min.	94 °C 30 s.	55 °C 40 s.	72 °C 40 s.	72 °C 7 min.	[30]
	Rev GTAAATGCACTTGCTTCAGGAC							
*mecA*	For GTA GAA ATG ACT GAA CGT CCG ATA A	310	94 °C 5 min.	94 °C 30 s.	50 °C 30 s.	72 °C 30 s.	72 °C 7 min.	[31]
	Rev CCA ATT CCA CAT TGT TTC GGT CTA A							
*hlg*	For GCCAATCCGTTATTAGAAAATGC	937	94 °C 5 min.	94 °C 30 s.	55 °C 40 s.	72 °C 1 min.	72 °C 10 min.	[32]
	Rev CCATAGACGTAGCAACGGAT							
*Staph 16sRNA*	For AAC TCT GTT ATT AGG GAA GAA CA	756	94 °C 5 min.	94 °C 30 s.	60 °C 40 s.	72 °C 1 min	72 °C 10 min	[33]
	Rev CCA CCT TCC TCC GGT TTG TCA CC							

### 2.7. Statistical Analysis

One hundred and ten equines were randomly selected from the study area using survey design and analysis software (survey toolbox program). The total number of equines in study were 13,000, with sample size of 0.85%. The statistical analyses were performed with SPSS 23 (IBM, Armonk, NY, USA). Numerical data are expressed as medians (ranges), while categorical data are expressed as numbers (%). For the assessment of risk factors, all hypothesized factors were categorized. Firstly, Chi-square tests and Fisher’s exact test for small sample sizes were conducted to assess the associations between various risk factors, including age, sex, breed, and season, and the frequency of isolation (percentage) of *S. aureus* isolated from ocular swabs. Furthermore, multivariate logistic regression analysis with forward conditional was applied. Fisher’s Exact Test was applied for breed and sex due to small sample size. The *p*-value, odds ratio (OR), and 95% confidence interval (CI 95%) were recorded to detect the associated risk factors of presence of *S. aureus*. For all statistical analyzes, variables at *p*-value < 0.05 were considered significant.

## 3. Results

### 3.1. Identification and Epidemiology of S. aureus in Healthy and Diseased Equine Eyes

The age range of examined equines in this study was 6 months to 22 years. In total, 47 putative *S. aureus* strains were isolated from individual animal eyes (one isolate per eye). Of them, 24 isolates were from 19 diseased animals and 23 isolates were from 23 healthy equines. Overall, the prevalence of *S. aureus* in eyes was 53.4%. The putative *S. aureus* strains were identified first by their characteristic appearance as yellow colonies with yellow zones on mannitol-phenol red agar due to the fermentation of mannitol with an acidic by-product. *S. aureus* was confirmed biochemically and by *nuc* gene PCR.

*S. aureus* was isolated in a higher proportion, 19 out of 33 (57.6%), from equines with diseased eyes as compared to those with healthy eyes (23 out of 77 (29.9%)) (Table 2). The frequency of *S. aureus* isolation also increased with age and season. The highest proportion of *S. aureus*-positive isolates was 31 out of the 42 (73.8%) isolates from 5- to 20-year-old horses, as compared to only 11 out of 42 (26.2%) *S. aureus*-positive isolates from horses that were less than 5 years old (Table 3). Regarding sex, out of the 42 *S. aureus*-positive cases, 37 (88.1%) were female, compared to 5 (11.9%) male horses. The multivariate logistic regression model revealed that season is a potential risk factor for prevalence of MRSA in equine eyes (*p*, 0.031; odds ratio: 2.5; confidence interval at 95%: 1.8–9.2). In terms of the effect of season, the highest proportion of isolates was the 32 (76.2%) recorded in the winter as compared to 10 (23.8%) isolates obtained in the spring (Table 3). With regard to antibiotic sensitivity, the *S. aureus* isolates from infected equine eyes were 100% sensitive to sulfamethoxazole, 75% to tetracycline, 75% to chloramphenicol, 70.83% to ampicillin, 33.33% to gentamycin, 25% to rifampicin, 8.33% to oxacillin, and 4.16% to cephalosporin. Meanwhile, these isolates were 90.48% resistant to oxacillin, 90.67% to cephalosporin, and 70.83% to rifampicin (Table 4). Clinical symptoms included watery-to-mucopurulent lacrimation, blepharospasm, conjunctival congestion, eye lid edema, corneal edema, and corneal opacity (Table 5).

Since *S. aureus* infection severity depends on some virulence factors, we used PCR to detect the presence of virulence and antibiotic resistance genes. PCR revealed that *mecA* was present in 100% of the oxacillin- and β-lactam-resistant strains. About 62.5% of *S. aureus* isolates possessed the *spa* (x region) gene, 100% had the *nuc* gene, and 56% contained the *hlg* gene. Moreover, 41.67% of the *S. aureus* isolates were found in both eyes of the same animal, while 58.33% were isolated from only one infected eye per animal (Table 5).

### 3.2. Sequencing and Phylogenetic Analysis of the Spa (x Region) Gene Fragment

**Figure 1:**
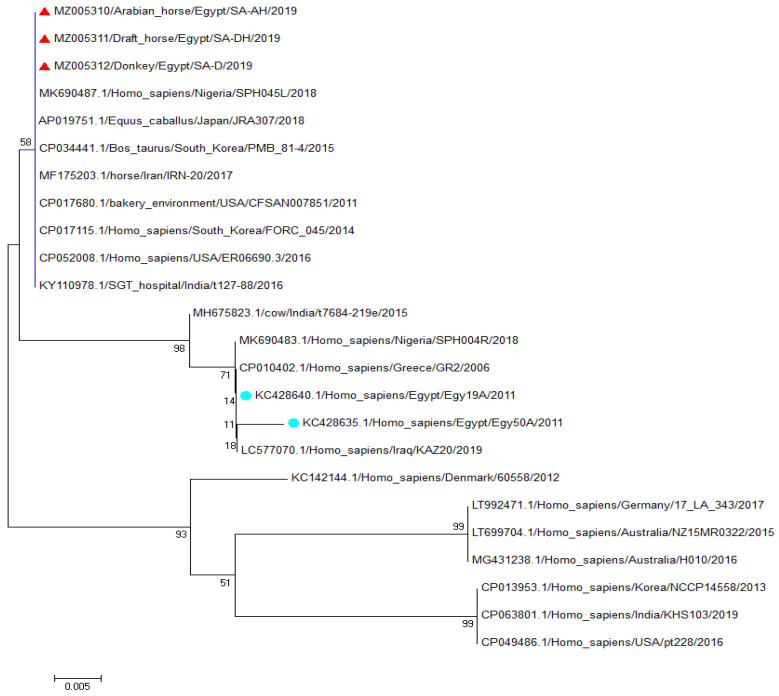
The phylogenetic neighbor-joining tree of three *S. aureus* strains. The alignment of nucleotides sequences for three selected samples shows that the strains identified in the present study (red triangles) are closely related to each other and to other *S. aureus* strains isolated from humans in Egypt (cyan circles).

Three samples were randomly selected from *S. aureus Spa* (x region)-gene-positive samples (one from each equine species). Sequences were analyzed against reference *S. aureus spa* (x region)-gene sequences in GenBank (Figure 1). The three identified strains of *S. aureus* strains (MZ005310/Arab horse/Egypt/SA-AH/2019, MZ005311/draft horse /Egypt/SA-DH/2019, and Z005312/Donkey/Egypt/SA-D/2019) identified in the present study were aligned with the Egyptian strain KC428640.1/Homo_sapiens/Egypt/Egy19A/2011 and KC428635.1/Homo_ sapiens/Egypt/Egy50A/2011, which were isolated from humans in Egypt (Figure 1). The *S. aureus Spa* (x region) from the Arabian horse, draft horse, and donkey were related to each other with 100% identity and shared a 97% identity with the KC428640.1/Homo_sapiens/Egypt/Egy19A/2011 *S. aureus* strain isolated from humans in Egypt. The identified *S. aureus* Arabian horse, draft horse, and donkey isolate *Spa* (x-region) genes had a 97.5% identity with KC428635.1/Homo_sapiens/Egypt/Egy50A/2011 (Supplemental Figure S1). These *S. aureus* isolates also had 100% identity with AP019751.1 /Equus_caballus /Japan/JRA307/2018, which was isolated from an equine in Japan, and MF175203.1 /horse/Iran/IRN-20/2017, which was isolated from a horse in Iran.

## 4. Discussion

Among the total number (110) of equine samples, the prevalence of ocular disease was 30%, which is consistent with a previous study in India [34] but higher than previously reported in Ethiopia (23.5%) [35]. The close rates in these countries could be due to similar windy and dusty environmental conditions, especially considering that the majority of ocular infections occurred in the winter in the current study. The rate of *S. aureus* isolation in the current study was much higher than a previous study in Poland with a rate of 9.8% [6]; this could be explained again by environmental differences and the increasing prevalence of *S. aureus* [36]. Differences in weather, environment, management, the presence of other infectious diseases, insect populations, and horse breeds are additional factors that may alter *S. aureus* prevalence. The higher percent (73.8%) of positive *S. aureus* isolates from horses aged 5–20 years is consistent with a previous study that demonstrated a high rate of *S. aureus* infection (87.8%) in horses older than 15 years [37].

The isolation of *S. aureus* from 19 out of 33 (57.6%) equines suffering ocular infection indicates that *S. aureus* is a major cause of ocular infection in equines. Similarly, *S. aureus* is reported to be a major cause of human eye infections [38], albeit at a lower rate (25%). This may be related to the unique structure of the equine eye structure, which is larger, more ovoid, and prominent from the sides, all of which make it more prone to damage by grass or dust that results in more ocular infection [39]. The *S. aureus* isolation rate of 29.9% from apparently healthy equine eyes might be the result of subclinical infections [37] or the presence of *S. aureus* as a member of the eye microbiota [4].

*S. aureus* has gained structural changes to the β-lactam target site through acquisition of the *mecA* gene, which enables resistance to oxacillin and methicillin. The expression of *mecA* in *S. aureus* enables it to synthesize a protein called the penicillin-binding protein, PBP2a, which decreases the binding affinity of β-lactams to MRSA strains [40]. The *mecA* gene is carried on the mec staphylococcal cassette chromosome (SCCmec), a mobile genetic element that is wide-spread in *S. aureus* due to extensive use of β-lactam antibiotics and related selective pressures. PBP2 is the main factor in the survival and success of *S. aureus* as a major pathogen in recent decades [41]. Oxacillin-resistant *S. aureus* is classified as MRSA according to the Institute of Clinical and Laboratory Standards (2005). In this study, the high resistance rate (91.67%) of isolated *S. aureus* to oxacillin indicates that most equine eye infections are probably MRSA. This assumption is boosted by the detection of the *mecA* gene in 100% of β-lactam-resistant *S. aureus* isolates [42]. Interestingly, the rate of MRSA among equine ocular infection in this study is higher than a previous study in India that reported an MRSA prevalence of 49% [43] and another study undertaken in China with a 52.8% MRSA prevalence [1]. In the region in which this study was conducted, the high rate of MRSA strains among ocular infections is serious and alarming and may reflect antibiotic overuse.

Of note, this study found a low resistance of *S. aureus* to tetracycline (25%), which contrasts with a previously reported resistance of 58% to tetracycline, especially among MRSA strains, and the suggested association of the *tet* gene with the SCCmec [44]. This difference can be attributed to in the geographical variation of MRSA in its prevalence and pattern of antibiotic sensitivity to antibiotics [45], and may also indicate the difference in the association between the *mecA* gene and the *tet* gene in the currently isolated *S. aureus*, in contrast to the previously assumed association between the oxacillin resistance genes and tetracycline resistance genes [44]. Importantly, the higher sensitivity of the MRSA strains isolated in this study to chloramphenicol (75%) and ampicillin (70.83%) as compared to those in other studies may reflect changes in *S. aureus*-antibiogram trends due to their lower rate of use in recent decades. Similarly, a recent report described changes in *Salmonella* antibiograms, including 60–80% sensitivity to chloramphenicol [46]. Additionally, another study in Egypt on *Salmonella* reported 92% sensitivity to chloramphenicol [47].

The detection of the *Spa* gene in 62.5% of *S. aureus* isolates indicates the highly pathogenic nature of these isolates, as the expression of this gene and its encoded protein A allow *S. aureus* to escape host immune system opsonization and phagocytosis [48] as well as inhibit host immune cell proliferation [49]. The *S. aureus Spa* (x region) is polymorphic and varies worldwide; its sequence is used as a typing method for clinical isolates of *S. aureus* [50]. This analysis revealed *spa* (x region) sequence conformity between *S. aureus* isolates from an Arabian horse, a draft horse, and a donkey, which indicates the local circulation of these isolates and its transmission among animals in the study region. This is consistent with the recently reported prevalence of *Spa* in some countries [51]. This may reflect the mandatory need for molecular typing of *S. aureus*, including *Spa*, across Egypt to better describe *S. aureus* type distribution. The variety of *S. aureus* clones in clinical settings of a cross-border region between the Netherlands and Germany was previously reported [52]. The current study also conducted a sequence analysis that revealed a close identity of the *spa* (x region) between *S. aureus* isolates from equine ocular infection and human isolates, which confirms its zoonotic nature. The phylogenic analysis also revealed that they were aligned together in the same clade (Figure 1) with at least 97% nucleotide similarity to the three equine samples (2.5–3% diversity, Supplemental Figure S1).

The presence of the *nuc* gene in 100% of the study isolates is also noteworthy as it encodes the thermostable nuclease enzyme that hydrolyzes host cell DNA and RNA, which devastates host tissue and facilitates pathogen dissemination, partially by avoiding neutrophil extracellular traps [53] and suppressing biofilm formation [54,55]. This gene confirmed the identification of our isolates as *S. aureus* since *nuc* is considered a useful marker for *S. aureus* detection [33]. The high rate of *mecA* gene expression and its associated high correlation with MRSA strains compared to the usual low rate of *nuc* detection in *S. aureus* isolates should be a reminder not to depend on one gene, especially *nuc*, to identify *S. aureus.* This observation agrees with the assumed probability of *S. aureus* misidentification if it only requires *nuc* gene detection, due to its variation [56]. The high expression rate (56%) of gamma-hemolysin (*hlg*) genes in *S. aureus* reflect their high virulence in equine ocular infections in Egypt. This rate is lower than the detection rate of *hlg* (87.5%) in community-associated MRSA encountered in children [57]. In particular, this study detected the *hlg* gene in most MR and four methicillin-sensitive *S. aureus* isolates, which supports previously reported data [58].

## 5. Conclusions

The results of this study demonstrated that most *S. aureus* isolates from clinically affected equine eyes are MRSA, as indicated by antibiograms and the detection of relevant genes. This research highlighted the local distribution and circulation of MRSA among equine ocular lesions in the studied area. This study also identified risk factors associated with the MRSA clinically affected equine eyes. Of these, the age and season have a significant effect while the breed and sex have non-significant effects. The phylogenetic analysis of *S. aureus* causing eye lesions in equines is of crucial importance as it indicates its zoonotic potential. In light of the current study, bacterial culture and antibiotic profiles and the molecular detection of MRSA isolates should be conducted in all locations within each country to generate procedures to limit the spread of multidrug-resistant genes. Future work on the zoonotic potential of equine ocular *S. aureus* should address whether the distribution of methicillin-resistant isolates from the same geographic region share the same sequences between equines and humans to confirm the zoonotic potential of this organism.

## Figures and Tables

**Table 2:** Frequency of *S. aureus* isolates from healthy and diseased equine eyes.

*S. aureus*	Healthy (*n* = 77)	Diseased (*n* = 33)	OR	*p*-Value	95% CI
			0.309	0.009	0.125–0.763
Positive	23 (29.9%)	19 (57.6%)			
Negative	54 (70.1%)	14 (42.4%)			

**Table 3:** Risk factors of *S. aureus* isolation from healthy and diseased equine eyes.

Risk Factor	*S. aureus*		OR	*p*-Value	95% CI
	Negative	Positive			
Age			3.368	0.003	1.412–7.960
<5	37 (54.4%)	11(26.2%)			
5–10	31 (45.6%)	31 (73.8%)			
Breed			7.053	0.06	0.07–0.6
Arabian	67 (98.5%)	38 (90.5%)			
Others	1 (1.5%)	4 (9.5%)			
Sex			2.869	0.05	1.0–1.798
Male	19 (27.9%)	5 (11.9%)			
Female	49 (72.1%)	37 (88.1%)			
Season			2.983	0.030	1.017–8.234
Winter	61 (89.7%)	32 (76.2%)			
Spring	7 (10.3%)	10 (23.8%)			

**Table 4:** Antimicrobial susceptibility for *S. aureus* isolates from diseased equine eyes.

Antimicrobial Sensitivity for *Staphylococcus aureus*			Antimicrobial Agent
Sensitive	Intermediate	Resistant	
24 (100%)	0 (0%)	0 (0%)	Sulfamethoxazole/trimethoprim (25 μg)
2 (8.33%)	0 (0%)	22 (91. 67%)	Oxacillin (1 μg)
18 (75%)	0 (0%)	6 (25%)	Tetracycline (30 μg)
6 (25%)	1 (4.16%)	17 (70.83%)	Rifamycin (5 μg)
17 (70.83%)	2 (8.33%)	5 (20.83%)	Ampicillin (10 μg)
8 (33.33%)	5 (20.83%)	11 (45.83%)	Gentamycin (10 μg)
1 (4.16%)	1 (4.16%)	22 (91.67%)	Cephalosporin (10 μg)
18 (75%)	1 (4.16%)	5 (20.83%)	Chloramphenicol (30 μg)

**Table 5:** Clinical signs, antibiotic resistance markers and virulence genes from *S. aureus* isolates of diseased equine eyes.

Case No.	Sample (Eye)	Equine Source	Age (in Years)	Clinical Signs	*spa* (x Region)	*nuc*	*mecA*	*hlg*
1	1(Right)	Arabian	0.5	corneal edema, eyelid edema, keratitis	−	+	+	+
2	2 (Left)	Arabian	6	mucopurulent discharge, corneal edema, eyelid edema, keratitis	−	+	+	+
3	3 (Right)	Arabian	8	conjunctivitis, watery lacrimation	−	+	+	−
4	4 Right)	Arabian	1.5	conjunctivitis	−	+	−	−
5	5 (Left)	Draft	1	mucopurulent discharge, corneal edema, eyelid edema, keratitis e	+	+	+	+
5	6 (Right)	Draft	1	mucopurulent discharge	−	+	+	+
6	7 (Left)	Arabian	2.5	corneal opacity	+	+	−	+
7	8 (Left)	Draft	4	conjunctivitis, watery lacrimation	+	+	+	−
7	9 (Right)	Draft	4	conjunctivitis, watery lacrimation	+	+	+	−
8	10 (Left)	Donkey	5	eyelid edema, mucopurulent discharge	+	+	−	+
9	11 (Right)	Arabian	9	conjunctivitis, watery lacrimation	+	+	+	−
9	12 (Left)	Arabian	9	conjunctivitis, watery lacrimation	+	+	+	−
10	13 (Left)	Arabian	5	conjunctivitis, watery lacrimation	+	+	+	−
10	14 (Right)	Arabian	5	conjunctivitis, watery lacrimation	+	+	+	−
11	15 (Left)	Arabian	11	corneal opacity	+	+	+	+
12	16 (Right)	Arabian	14	conjunctivitis, watery lacrimation	+	+	+	−
13	17 (Right)	Donkey	13	eyelid edema, keratitis, mucopurulent discharge	+	+	+	+
14	18 (Left)	Arabian	6	corneal edema, eyelid edema, keratitis	+	+	+	+
15	19 (Right)	Donkey	9	eyelid edema, keratitis, mucopurulent discharge	+	+	+	+
16	20 (Left)	Arabian	12	lacrimation, conjunctivitis	−	+	+	−
17	21 (Left)	Arabian	7	corneal edema, eyelid edema, keratitis, mucopurulent discharge	−	+	+	+
18	22 (Left)	Donkey	7	eyelid edema, keratitis, mucopurulent discharge	+	+	+	+
19	23 (Left)	Arabian	9	eyelid edema, keratitis, mucopurulent discharge	−	+	+	+
19	24 (Right)	Arabian	9	eyelid edema, keratitis, mucopurulent discharge	−	+	+	+

## Data Availability

The data presented in this study are openly available on the PubMed database.

## Supplementary Materials

The following supporting information can be downloaded at: https://www.mdpi.com/article/10.3390/antibiotics11020221/s1, Figure S1: Diversity percent between *S. aureus* strains identified in this study and other strains from GenBank. Diversity was calculated by MEGA X software.
