# Supplementary material for: The Prevalence and Molecular Biology of Staphylococcus aureus Isolated from Healthy and Diseased Equine Eyes in Egypt
Source: Antibiotics (Basel). 2022 Feb 10;11(2):221. doi: 10.3390/antibiotics11020221 (PMC8868267; doi:10.3390/antibiotics11020221)
Supplement: Supplementary file 1 [file antibiotics-11-00221-s001.zip › antibiotics-1504931-supplementary.pdf]

antibiotics

MDPI

Supplementary

The Prevalence and Molecular Biology of *Staphylococcus aureus* Isolated from Healthy and Diseased Equine Eyes in Egypt

Amin Tahoun [1,*], Helmy K. Elnafarawy [2], Hanem El-Sharkawy [3], Amira M. Rizk [4], Mohammed Alorabi [5], Ahmed M. El-Shehawi [5], Mohamed A. Youssef [2], Hussam M. M. Ibrahim [2] and Sabry El-Khodery [2]

1 Department of Animal Medicine, Faculty of Veterinary Medicine, Kafrelshkh University, Kafrelsheikh 33511, Egypt
2 Department of Internal Medicine and Infectious Diseases, Faculty of Veterinary Medicine, Mansoura University, Mansoura 35516, Egypt; helmykamal@mans.edu.eg (H.K.E.); mohamed.youssef@mans.edu.eg (M.A.Y.); dr_hussamhabosha@yahoo.com (H.M.M.I.); khodery@mans.edu.eg (S.E.)
3 Department of Poultry and Rabbit Diseases, Faculty of Veterinary Medicine, Kafrelsheikh University, Kafrelsheikh 33511, Egypt; hanem_amin@yahoo.com
4 Department of Bacteriology, Mycology and Immunology, Faculty of Veterinary Medicine, Benha University, Benha 13518, Egypt; dr_az80@yahoo.com
5 Department of Biotechnology, College of Science, Taif University, P.O. Box 11099, Taif 21944, Saudi Arabia; maorabi@tu.edu.sa (M.A.); a.elshehawi@tu.edu.sa (A.M.E.-S.)
* Correspondence: amin12_veta@yahoo.com

	1	2	3	4	5	6	7	8	9	10	11	12	13	14	15	16	17	18	19	20	21	22	23	24
1- MZ005310/Arabian_horse/Egypt/SA-AH/2019																								
2- MZ005311/Draft_horse/Egypt/SA-DH/2019	0.0%																							
3- MZ005312/Donkey/Egypt/SA-D/2019	0.0%	0.0%																						
4- MK690487.1/Homo_sapiens/Nigeria/SPH045L/2018	0.0%	0.0%	0.0%																					
5- AP019751.1/Equus_caballus/Japan/JRA307/2018	0.0%	0.0%	0.0%	0.0%																				
6- CP034441.1/Bos_taurus/South_Korea/PMB_81-4/2015	0.0%	0.0%	0.0%	0.0%	0.0%																			
7- MF175203.1/horse/Iran/IRN-20/2017	0.0%	0.0%	0.0%	0.0%	0.0%	0.0%																		
8- CP017680.1/bakery_environment/USA/CFSAN007851/201	0.0%	0.0%	0.0%	0.0%	0.0%	0.0%	0.0%																	
9- CP017115.1/Homo_sapiens/South_Korea/FORC_045/201	0.0%	0.0%	0.0%	0.0%	0.0%	0.0%	0.0%	0.0%																
10- CP052008.1/Homo_sapiens/USA/ER06690.3/2016	0.0%	0.0%	0.0%	0.0%	0.0%	0.0%	0.0%	0.0%	0.0%															
11- KY110978.1/SGT_hospital/India/t127-88/2016	0.0%	0.0%	0.0%	0.0%	0.0%	0.0%	0.0%	0.0%	0.0%	0.0%														
12- MH675823.1/cow/India/t7684-219e/2015	2.0%	2.0%	2.0%	2.0%	2.0%	2.0%	2.0%	2.0%	2.0%	2.0%	2.0%													
13- MK690483.1/Homo_sapiens/Nigeria/SPH004R/2018	2.5%	2.5%	2.5%	2.5%	2.5%	2.5%	2.5%	2.5%	2.5%	2.5%	2.5%	0.5%												
14- CP010402.1/Homo_sapiens/Greece/GR2/2006	2.5%	2.5%	2.5%	2.5%	2.5%	2.5%	2.5%	2.5%	2.5%	2.5%	2.5%	0.5%	0.0%											
15- KC428640.1/Homo_sapiens/Egypt/Egy19A/2011	2.5%	2.5%	2.5%	2.5%	2.5%	2.5%	2.5%	2.5%	2.5%	2.5%	2.5%	0.5%	0.0%	0.0%										
16- KC428635.1/Homo_sapiens/Egypt/Egy50A/2011	3.0%	3.0%	3.0%	3.0%	3.0%	3.0%	3.0%	3.0%	3.0%	3.0%	3.0%	1.0%	0.5%	0.5%	0.5%									
17- KC142144.1/Homo_sapiens/Denmark/60558/2012	3.5%	3.5%	3.5%	3.5%	3.5%	3.5%	3.5%	3.5%	3.5%	3.5%	3.5%	4.6%	5.1%	5.1%	5.1%	5.6%								
18- MG431238.1/Homo_sapiens/Australia/H010/2016	5.1%	5.1%	5.1%	5.1%	5.1%	5.1%	5.1%	5.1%	5.1%	5.1%	5.1%	7.3%	7.8%	7.8%	7.8%	8.4%	3.5%							
19- CP013953.1/Homo_sapiens/Korea/NCCP14558/2013	5.1%	5.1%	5.1%	5.1%	5.1%	5.1%	5.1%	5.1%	5.1%	5.1%	5.1%	7.3%	7.9%	7.9%	7.9%	8.5%	4.7%	5.1%						
20- LT992471.1/Homo_sapiens/Germany/17_LA_343/2017	5.1%	5.1%	5.1%	5.1%	5.1%	5.1%	5.1%	5.1%	5.1%	5.1%	5.1%	7.3%	7.8%	7.8%	7.8%	8.4%	3.5%	0.0%	5.1%					
21- LT699704.1/Homo_sapiens/Australia/NZ15MR0322/2015	5.1%	5.1%	5.1%	5.1%	5.1%	5.1%	5.1%	5.1%	5.1%	5.1%	5.1%	7.3%	7.8%	7.8%	7.8%	8.4%	3.5%	0.0%	5.1%	0.0%				
22- CP063801.1/Homo_sapiens/India/KHS103/2019	5.1%	5.1%	5.1%	5.1%	5.1%	5.1%	5.1%	5.1%	5.1%	5.1%	5.1%	7.3%	7.9%	7.9%	7.9%	8.5%	4.7%	5.1%	0.0%	5.1%	5.1%			
23- LC577070.1/Homo_sapiens/Iraq/KAZ20/2019	2.5%	2.5%	2.5%	2.5%	2.5%	2.5%	2.5%	2.5%	2.5%	2.5%	2.5%	0.5%	0.0%	0.0%	0.0%	0.5%	5.1%	7.8%	7.9%	7.8%	7.8%	7.9%		
24- CP049486.1/Homo_sapiens/USA/pt228/2016	5.1%	5.1%	5.1%	5.1%	5.1%	5.1%	5.1%	5.1%	5.1%	5.1%	5.1%	7.3%	7.9%	7.9%	7.9%	8.5%	4.7%	5.1%	0.0%	5.1%	5.1%	0.0%	7.9%	

Figure S1. Diversity percent between *S. aureus* strains identified in this study and other strains from GenBank. Diversity was calculated by MEGA X software.
